# Whole Stomach 3D Reconstruction and Frame Localization From Monocular Endoscope Video

**DOI:** 10.1109/JTEHM.2019.2946802

**Published:** 2019-10-18

**Authors:** Aji Resindra Widya, Yusuke Monno, Masatoshi Okutomi, Sho Suzuki, Takuji Gotoda, Kenji Miki

**Affiliations:** 1 Department of Systems and Control EngineeringSchool of EngineeringTokyo Institute of Technology 13290 Tokyo152-8550Japan; 2 Division of Gastroenterology and HepatologyDepartment of MedicineNihon University School of MedicineTokyo101-8309Japan; 3 Department of Internal MedicineTsujinaka Hospital KashiwanohaKashiwa277-0871Japan

**Keywords:** Gastric endoscopy, monocular endoscope, stomach, 3D reconstruction, structure-from-motion

## Abstract

Gastric endoscopy is a common clinical practice that enables medical doctors to diagnose various lesions inside a stomach. In order to identify the location of a gastric lesion such as early cancer and a peptic ulcer within the stomach, this work addresses to reconstruct the color-textured 3D model of a whole stomach from a standard monocular endoscope video and localize any selected video frame to the 3D model. We examine how to enable structure-from-motion (SfM) to reconstruct the whole shape of a stomach from endoscope images, which is a challenging task due to the texture-less nature of the stomach surface. We specifically investigate the combined effect of chromo-endoscopy and color channel selection on SfM to increase the number of feature points. We also design a plane fitting-based algorithm for 3D point outliers removal to improve the 3D model quality. We show that whole stomach 3D reconstruction can be achieved (more than 90% of the frames can be reconstructed) by using red channel images captured under chromo-endoscopy by spreading indigo carmine (IC) dye on the stomach surface. In experimental results, we demonstrate the reconstructed 3D models for seven subjects and the application of lesion localization and reconstruction. The methodology and results presented in this paper could offer some valuable reference to other researchers and also could be an excellent tool for gastric surgeons in various computer-aided diagnosis applications.

## Graphical abstract

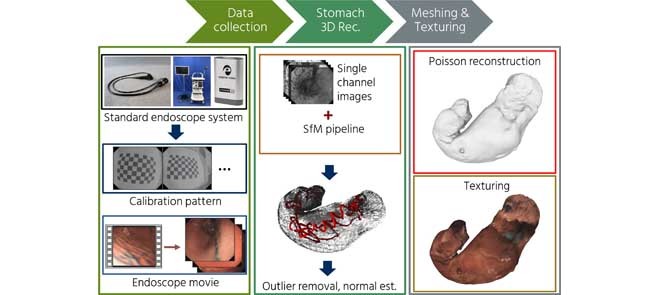

## I. Introduction

Gastric endoscopy is a well-adopted procedure that enables medical doctors to diagnose various lesions inside a stomach. The accurate localization of a malignant lesion within the global view (i.e., global 3D structure) of the stomach is crucial for gastric surgeons to make a clinical decision of the operative procedure for early cancer. However, it is difficult for gastric surgeons to recognize the lesion’s 3D location from 2D endoscope images captured by other endoscopists due to the limited viewing angle of an endoscope camera, the lack of depth perception, and the uncertainty of endoscope 3D poses relative to a stomach surface. Therefore, the lesion location is often confirmed by double contrast barium radiography [1]. However, morphological evaluation such as the barium study sometimes causes difficulty for gastric surgeons in identifying flat malignant lesions. Recently, 3D computed tomography (CT) gastrography was developed for the detection of gastric abnormalities [2]. Although the 3D CT gastrography can provide an accurate stomach 3D model, it is still difficult to identify and localize the lesion only from morphological information, since it does not embedded color texture information. If the 3D model of a whole stomach can be reconstructed from a standard endoscope video, the location of a malignant lesion can be easily identified using the visual color information in addition to the 3D morphological information, which should be very valuable for gastric surgeons.

Previous studies have shown that 3D endoscope systems, such as a stereo endoscope [3] and a time-of-flight endoscope system [4], have advantages over traditional 2D endoscopes in applications such as laparoscopic computer-aided surgery [5], endoscopic surface imaging [6], and real-time visual odometry [7]. Nevertheless, those 3D endoscopes are not widely available and the 2D counterpart is still the mainstream.

There are also many existing vision-based methods to reconstruct the 3D surface of a target organ while estimating the endoscope poses from a monocular endoscope video (see [8]–[10] for the surveys). The methods are ranging from shape-from-shading (SfS) [11]–[13], visual simultaneous localization and mapping (SLAM) [14]–[17], and structure-from-motion (SfM) [18]–[22]. However, most of existing works only have demonstrated the reconstruction result of a partial surface of the target organ, which is not sufficient for our localization purpose.

In this paper, we adopt an off-line SfM pipeline and examine how to enable SfM to reconstruct the 3D model of a whole stomach from a standard monocular endoscope video, aiming at the 3D lesion localization. We specifically investigate the combined effect of chromo-endoscopy and color channel selection on SfM to increase the number of feature points and achieve better reconstruction quality and completeness. To improve an initial SfM result, we also develop a 3D point outlier removal algorithm based on local plane fitting with random sampling consensus (RANSAC) [23]. The color-textured mesh model is then generated from the outlier-removed point cloud. We finally present our frame localization and local reconstruction pipeline based on a selected reference frame (e.g., a frame with a lesion) to identify the 3D location of the frame and obtain a more detailed reconstruction result around the frame. To the best of our knowledge, this is the first paper to report successful 3D reconstruction of a whole stomach from a standard monocular endoscope video and apply the reconstructed stomach 3D model to visualize the color details of a mucosal surface by texture mapping from the endoscope images.

This paper is an extended version of the paper published in [24]. In this paper, we offer more detailed discussion on input image processing and point cloud processing based on an improved outlier removal algorithm. We also present some new results for additional subjects and an additional potential application of lesion localization and reconstruction.

The rest of this paper is organized as follows. Section II presents the data collection and pre-processing procedure and the 3D reconstruction pipeline. Section III reports experimental results. Section IV provides the discussion of the result and concludes the paper.

## II. Method and Procedure

In this section, we describe the data collection procedure and the 3D reconstruction method. We first explain our endoscopy hardware setup and the captured video sequences information (Section II-A). Then, we explain the data pre-processing to extract data for SfM inputs (Section II-B). Then, we detail each component of our 3D reconstruction pipeline, including point cloud reconstruction by SfM (Section II-C), 3D point outliers removal (Section II-D), and mesh and texture generation (Section II-E). We finally present our frame localization and local reconstruction pipeline (Section II-F). The overall flow of our processing pipeline is shown in Fig 2(a).

**FIGURE 1.**
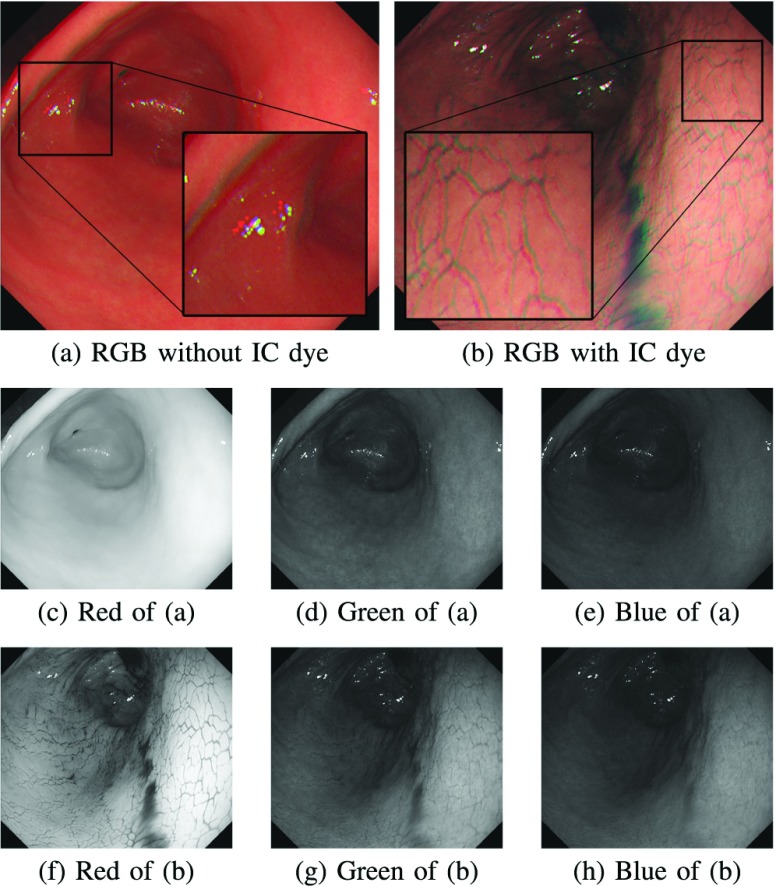
Examples of endoscope images captured (a) without the IC dye and (b) with the IC dye. The color channel misalignment is observed in (a) and (b). The images (c) to (h) are six single-channel images extracted from (a) and (b). We can observe that the IC dye adds textures on the stomach surface, especially in the red channel (f).

**Fig. 2.**
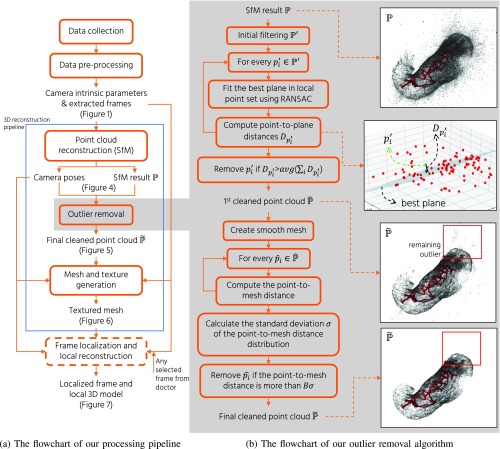
The flowchart of (a) our overall processing pipeline and (b) our outlier removal algorithm. We also show the point cloud result of Subject A in each step of the outlier removal. See Section II-D for detailed explanation of the algorithm.

### A. Data Collection

We captured the endoscope videos using a standard monocular endoscope system. We used an Olympus IMH-20 image management hub coupled with a GIF-H290 scope. To prevent any compression and unwanted artifacts such as image interlacing, we used an Ephipan DVI2USB 3.0 video grabber to capture unprocessed data from the image management hub. The video was saved as an AVI format at 30 frames per second with $1156\times 1004$ effective resolution, as shown in Fig. 1.

The videos were captured on seven subjects undergoing general gastrointestinal endoscopy under sedation with midazolam. Ten milligrams of scopolamine butylbromide was also used during endoscopy in order to prevent bowel and stomach movement. As shown in Fig. 1(a) and 1(b), each video contains two image sequences captured without and with spraying the indigo carmine (IC) blue color dye onto the stomach surface as chromo-endoscopy [25]. The IC dye is the most commonly used dye to enhance the surface visualization. For the IC dye, we used C_16_H_8_N_2_Na_2_O_8_S_2_ manufactured by Daiichi Sankyo Company, Limited, Tokyo, Japan. In addition to general gastrointestinal endoscopy, five minutes additional time is needed to allow the IC dye to cover all the stomach surface and to capture the entire stomach surface. However, there is no additional sedation needed.

**Ethics.** This study was conducted in accordance with the Declaration of Helsinki. The Institutional Review Board at Nihon University Hospital approved the study protocol on March 8, 2018, before patient recruitment. Informed consent was obtained from all patients before they were enrolled. This study was registered with the University Hospital Medical Information Network (UMIN) Clinical Trials Registry (Identification number: UMIN000031776) on March 17, 2018. This study was also approved by the research ethics committee of Tokyo Institute of Technology, where 3D reconstruction experiments were conducted.

### B. Data Pre-Processing

The data pre-processing is performed to estimate camera intrinsic parameters and to extract input images for SfM. This process includes camera calibration, frame extraction, color channel separation, and duplicated frame removal as follows.

An endoscope camera generally uses an ultra-wide lens to provide a large angle of view. As a trade-off, the ultra wide lens introduces a strong visual distortion and produces images with convex non-rectilinear appearance. If the distortion is not fixed, it can lead to incorrectly estimated 3D points. Therefore, camera calibration is needed to obtain the camera intrinsic parameters such as focal length, projection center, and distortion parameters. For the camera calibration purpose, we capture images of a planar checkerboard pattern from multiple orientations. We then use the captured planar checkerboard pattern images and a fish-eye camera model for the camera calibration [26]. The acquired camera intrinsic parameters are used to optimize the 3D points and the endoscope camera poses in SfM and to correct the image’s distortion. The camera calibration is required only once for each endoscope.

In the input images extraction process, we first extract all RGB frames from each video. Then, we extract two kinds of image sequences from each video, where the first one consists of the images captured under conventional endoscopy without the IC dye (Fig. 1(a)), while the second one consists of the images captured under chromo-endoscopy with the IC dye (Fig. 1(b)). After in-depth inspection, we find that there are many color artifacts in the RGB images caused by color channel misalignment as can be seen in Fig. 1(a) and 1(b). To minimize the effect of the artifacts, we decide to separate each RGB image into R, G, and B images and use each single-channel image sequence as an SfM input. In total, we use six single-channel image sequences (see Fig. 1(c) to 1(h) for the examples of each single-channel image) and investigate the combined effect of chromo-endoscopy and color channel selection on the SfM quality.

We also remove any duplicated frames that have almost no difference between successive frames. We observe that, in single-channel image sequences, there are frames that have very similar appearance compared to its successive frame. We presume that the imperfection of the capturing hardware leads to this problem. Since such duplicated frames are redundant and only add complexity to SfM, especially in feature matching and feature triangulation steps, we remove the duplicated frames as follows.

Let ${\mathbf I}_{t}$ and ${\mathbf I}_{t+1}$ be a reference frame and its successive frame, respectively. We take their absolute image difference, ${\mathbf I}_{d} = |{\mathbf I}_{t} - {\mathbf I}_{t+1}|$, and calculate the ratio of the number of pixels having non-zero values (i.e., the pixels having different pixel values between the frames) to the total number of pixels in ${\mathbf I}_{d}$. If the ratio is less than a threshold, $\phi $, we remove ${\mathbf I}_{t+1}$ as a duplicated frame and continue to compare ${\mathbf I}_{t}$ with its next successive frames (i.e., ${\mathbf I}_{t+2}$, ${\mathbf I}_{t+3}$, and so on) until finding a non-duplicated frame of ${\mathbf I}_{t}$. This process is repeated while updating the reference frame, where a new reference frame is the non-duplicated frame of the current reference frame.

### C. Point Cloud Reconstruction (SFM)

The point cloud reconstruction follows a general flow of an SfM pipeline [27], [28], assuming that the stomach has minimum movements. The algorithm starts with extracting features from the single-channel input frames and matching the extracted features, and then followed by the endoscope camera poses estimation and the feature points triangulation in parallel. These processing steps generate a sparse point cloud of the stomach and estimated each frame’s pose based on the endoscope motion.

We use SIFT [29] for feature detection and description and exhaustively search to find the feature correspondences among all input frame pairs. Since the feature correspondences search is solely based on image appearance, there is no guarantee that every feature correspondence maps the same point in the stomach. Thus, RANSAC [23] is applied to geometrically verify the correspondences between every input frame pairs. The feature triangulation step then starts from a carefully selected initial frame pair by performing two-view reconstruction [27]. Then, it incrementally registers new frames by solving the perspective-n-point (PnP) problem to estimate the newly registered frame’s pose [23]. This process leverages the connection between already triangulated 3D points and feature points in the newly registered frame. After the newly registered frame’s pose is estimated, new 3D points can be added to the scene by triangulating feature points as long as there is at least one feature correspondence in other frames. Finally, global bundle adjustment is performed to optimize the 3D points and the camera poses while minimizing the reprojection errors using the all feature correspondences and the pre-estimated camera intrinsic parameters [30].

### D. Outliers Removal

Since the initial point cloud from SfM, $\mathbb {P}$, contains many outlier points, as can be seen in $\mathbb {P}$ of Fig. 2(b), we need to remove outliers to produce a clean point cloud. Our previously proposed algorithm [24] tries to clean the SfM result by downsampling the initial point cloud to a fixed number of 3D points and remove outliers using a statistical approach. Unfortunately, this method not only produces a low-resolution mesh, but also leaves many outlier points. Because of that, we propose an improved outlier removal algorithm based on local plane fitting with RANSAC [23].

Figure 2(b) shows the overall flow of our outlier removal algorithm. Inspired by [31], our algorithm starts by filtering out isolated outlier points, which are the points far from any other points, based on the diagonal size, $r$, of the bounding box of $\mathbb {P}$. We calculate the nearest neighbour point-to-point distance of every point and removed the point if the distance to its nearest neighbour was more than $Ar$, where $A$ is an empirically determined parameter, resulting in an initially filtered point cloud, $\mathbb {P}'$. We then recalculate the bounding box size, $r$, after the initial filtering.

To preserve local details of the stomach surface, we treat outliers removal as a local plane fitting problem. For each remaining point, $p'_{i} \in \mathbb {P}'$, we search its neighborhood points inside a radius, $r$, to form a local point set, $\mathbb {P}'_{p'_{i}}$, for the local plane fitting. If there are more than 100 neighboring points, we only use the 100 nearest neighbor points. In addition, to ensure that there are enough points for the plane fitting, we remove the points having less than $M$ neighborhood points as outliers.

We then apply RANSAC [23] to fit the best plane for each local point set, $\mathbb {P}'_{p'_{i}}$, based on three random points. Then, we calculate the distance, $D_{p'_{i}}$, between the center point, $p'_{i}$, and the fitted best plane, as illustrated in the second top figure in Fig. 2(b). We then remove the point if the point-to-plane distance, $D_{p'_{i}}$, is more than the average distance, $ave(\Sigma _{i} D_{p'_{i}})$, to obtain a first cleaned point cloud, $\hat {\mathbb {P}}$.

Unfortunately, the first cleaned point cloud still contains remaining outliers, as can be seen in $\hat {\mathbb {P}}$ of Fig. 2(b). To further clean the point cloud, we construct a very smooth mesh from $\hat {\mathbb {P}}$ and measure the distance between every point, $\hat {p}_{i} \in \hat {\mathbb {P}}$, to the smooth mesh. We then calculate the standard deviation, $\sigma $, from the point-to-mesh distance distribution and filter out the point if the point-to-mesh distance is more than $B\sigma $, where $B$ is an empirically determined parameter. After the above processing steps, we obtain a final cleaned point cloud, $\tilde {\mathbb {P}}$, where outlier points are effectively removed as shown in $\tilde {\mathbb {P}}$ of Fig. 2(b).

### E. Mesh and Texture Generation

Given a final cleaned point cloud, $\tilde {\mathbb {P}}$, we then generate a triangle mesh. We firstly estimate the normal of each inlier 3D point based on its 100 nearest neighbour points [32]. Each estimated normal is further refined using the related endoscope camera poses to prevent it from pointing outward. Additionally, we apply normal smoothing to the refined normals. Then, the mesh is reconstructed by Poisson surface reconstruction based on the estimated normal for each point [33].

To add more visual detail and functionality, we also apply a color texture from the RGB images to the generated mesh based on the registered endoscope cameras in the SfM step. For each triangle mesh, we obtain a list of visible cameras as the possible candidates for texturing. Then, the frame in the candidates list that have the closest and the most orthogonal angle to the corresponding triangle mesh is chosen as a reference image. After that, optimization based on the triangle-to-camera angle and distance is applied to make sure that there is no isolated triangle mesh. Next, patches that correspond to every connected triangle having the same reference image are extracted and packed into a single texture space. Finally, a color-textured mesh model is created by mapping the patch in the texture space to the corresponding triangle in the generated mesh [34].

### F. Frame Localization and Local Reconstruction

Frame localization is performed using the estimated endoscope camera poses and the generated mesh. Using the localized frames, we can visualize a manually selected frame containing a gastric lesion, which is very useful for doctors to identify the lesion location within the global 3D structure of the stomach. We believe in that, for diagnosis applications, it is also very useful if we can provide a detail local 3D model of an interesting region in addition to the whole stomach 3D model. Thus, we subsequently present a local reconstruction pipeline based on a selected reference frame containing an interesting region such as a lesion.

We first retrieve top $N$ most similar images to the selected reference RGB image among the input RGB sequence using NetVLAD [35] with the pre-trained convolutional neural network (CNN) provided by the authors. NetVLAD first extracts the CNN-based features from all input images. It then describes each image, ${\mathbf I}_{t}$, with a feature vector, $ {f}({\mathbf I}_{t})$, by aggregating the extracted CNN features. Then, the similarity between the reference image, ${\mathbf I}_{r}$, and other images in the sequence can be measured by calculating the Euclidean distance of the corresponding aggregated feature vectors as $d = || {f}({\mathbf I}_{r}) -  {f}({\mathbf I}_{t})||$. We then input the single-channel images of the retrieved $N$ images by NetVLAD to the 3D reconstruction pipeline to obtain the mesh of the local interesting region. We finally apply the texture from the original RGB images to the previously obtained mesh using the single-channel images.

## III. Results

### A. Implementation Details

We performed the endoscope camera calibration using the OpenCV camera calibration library [36]. The SfM pipeline was implemented using Colmap [27]. We set as $\phi =0.6$ for the duplicated frames removal and set as $A=0.05$, $M=80$, and $B=5$ for outliers removal to generate a triangle mesh. For the local reconstruction, we set $N=100$ to retrieve 100 most similar images by NetVLAD [35]. We applied screened Poisson reconstruction [33] for triangle mesh generation. For the texturing purpose, we applied the texturing function from Meshlab [34].

### B. Point Cloud and Outlier Removal Results

**FIGURE 3.**
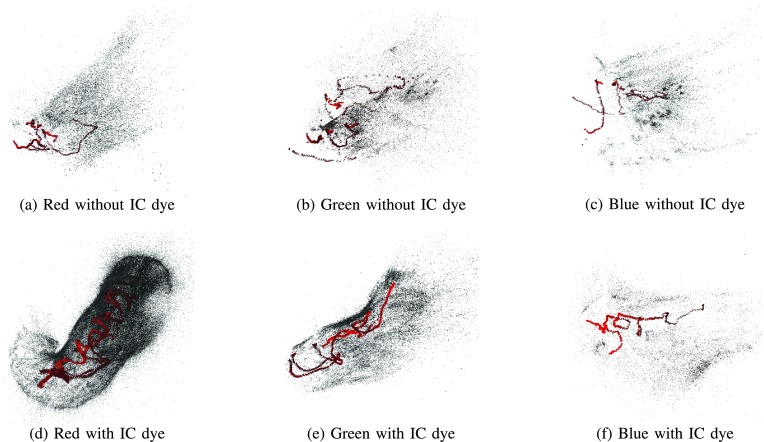
The initial 3D point cloud results on Subject A. The gray dots represent the reconstructed 3D points and the red pyramids represent the estimated endoscope poses. There is a significant difference between the cases with and without the IC dye. Only a sparse and small part of the stomach can be reconstructed in the case of without the IC dye. Moreover, because of the texture-less surface in the case of without the IC dye, the integrity of the structure is not sufficient. On the other hand, the whole stomach can be reconstructed using the red channel with the IC dye.

Figure 3 shows the initial 3D point cloud results by SfM on Subject A, which are reconstructed using different color channels of the cases without and with the IC dye. In general, the channels with the IC dye (Fig. 3(d)–3(f)) give a more complete reconstruction result compared to the channels without the IC dye (Fig. 3(a)–3(c)). In the case without the IC dye, each channel’s result fail to show any structural integrity. In the case with the IC dye, the red channel result has the whole shape of the stomach, while the green and the blue channel results barely represent the whole stomach shape. Among the RGB channels, the red channel gives the most complete and densest result. Some parts of the stomach could be reconstructed using the green channel, while the result of the blue channel was hardly interpretable.

Table 1 shows the objective evaluation of the initial 3D point cloud results on all seven subjects. The first and second rows for each category show the original number of frames extracted from each sequence and the number of remaining frames after the duplicated frames removal, respectively. We can confirm that many frames are unexpectedly duplicated. Those duplicated frames could effectively be removed by our algorithm. The third and fourth rows show the number of reconstructed frames and that of 3D points. These results show that the number of 3D points is generally higher when the IC dye is present. We also notice that the average observation (shown in the fifth row), which represents the per-image average number of the 2D feature points that can be triangulated into the 3D points, is generally increased when the IC dye exists. In addition, the percentage of reconstructed frames over input frames is significantly increased by using the IC dye. Among all the results, the red channel with the IC dye gives the best result, where more than 90% of the frames could be reconstructed for all subjects. When the IC dye is not present, the green channel gives the best result.

**TABLE 1:** The Objective Evaluation of the Initial Point Cloud Results Using Each Color Channel Without and With the IC Dye

		Subject A			Subject B			Subject C			Subject D		
		Red	Green	Blue	Red	Green	Blue	Red	Green	Blue	Red	Green	Blue
Without IC dye	Input frames	1680	1680	1680	1251	1251	1251	4501	4501	4501	1251	1251	1251
	After duplicate removal	1120	1115	1096	734	729	731	2959	2790	2977	831	827	821
	Reconstructed frames	417 (37.2%)	659 (59.1%)	385 (35.1%)	177 (24.1%)	226 (31.0%)	138 (18.9%)	1064 (36.0%)	1142 (40.9%)	946 (31.8%)	104 (12.5%)	449 (54.2%)	96 (11.6%)
	3D points	16343	30163	11999	8252	15319	3117	47960	79733	40073	2085	21202	2517
	Average observation	288	315	202	385	509	158	329	467	283	127	336	179
With IC dye	Input frames	2200	2200	2200	3500	3500	3500	3501	3501	3501	2251	2251	2251
	After duplicate removal	1470	1462	1449	2329	2331	2319	2327	2304	2323	1472	1471	1465
	Reconstructed frames	1470 (100%)	528 (36.1%)	394 (27.2%)	2246 (96.4%)	1488 (63.8%)	335 (15.3%)	2297 (98.7%)	891 (38.7%)	361(15.5%)	1382 (93.8%)	901 (61.3%)	305 (20.8%)
	3D points	323612	47711	14866	515762	100114	12035	727954	152223	14022	238938	53771	6374
	Average observation	1999	671	229	1971	503	207	2656	1195	221	1484	431	123
			Subject E			Subject F			Subject G				
			Red	Green	Blue	Red	Green	Blue	Red	Green	Blue		
	Without IC dye	Input frames	3000	3000	3000	4501	4501	4501	2000	2000	2000		
		After duplicate removal	1993	2007	1980	2980	2975	3000	1293	1297	1311		
		Reconstructed frames	82 (4.1%)	148 (7.3%)	136 (6.8%)	207 (6.9%)	687 (23.1%)	497(16.6%)	441 (34.1%)	1293 (99.6%)	888 (67.7%)		
		3D points	2574	8006	7637	5740	70610	22764	13946	94873	53295		
		Average observation	204	439	399	173	670	282	240	544	435		
	With IC dye	Input frames	2300	2300	2300	2251	2251	2251	2100	2100	2100		
		After duplicate removal	1534	1534	1535	1483	1489	1476	1537	1506	1504		
		Reconstructed frames	1498 (97.6%)	1231 (80.2%)	144 (9.3%)	1481 (99.8%)	1249 (83.9%)	567 (38.4%)	1534 (99.8%)	1506 (100%)	1475 (90.1%)		
		3D points	559180	127461	5932	731070	359418	49982	743575	394049	184156		
		Average observation	2906	688	226	4188	1988	487	4103	1956	848		

The above subjective and objective evaluation consistently shows that the red channel with the IC dye gives the best result. As shown in Fig. 1(c) to 1(h), this is because the red channel leverages the effect of the IC dye more than the other channels. In Fig. 1(f), many textures, from which many distinctive feature points can be extracted, are apparent in the red channel. When the IC dye is not used, the green channel has better contrasts compared to the other channels. The blue channel is the least preferable among those three channels for both cases without and with the IC dye.

**FIGURE 4.**
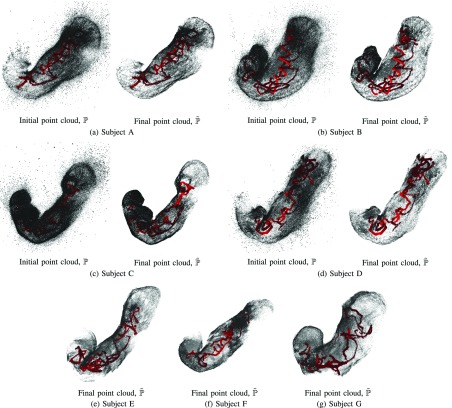
The point cloud results when using the red channel images with the IC dye. The red pyramids represent the estimated endoscope camera poses and the gray points represent the 3D points. For Subject A to D, we show the comparison between the initial point cloud, $\mathbb {P}$, and the final result of our outlier removal algorithm, $\tilde {\mathbb {P}}$, while only the final results are shown for Subject E to G due to the space limitation. From these results, we can confirm that our outlier removal algorithm can produce a clean point cloud. In some subjects, some parts have a noticeable hole. It is because some parts of the stomach are not captured in the endoscope video.

Figure 4 shows the point cloud result when using the red channel images with the IC dye as the SfM input. For Subject A to D, we show the comparison between the initial point cloud, $\mathbb {P}$, and the final outlier removed point cloud, $\hat {\mathbb {P}}$. The results demonstrate that the outputs of our proposed outlier removal algorithm are free from apparent outliers. It is also observed that our outlier removal algorithm preserves the structure of the initial point cloud. In some subjects, some parts have a noticeable hole. It is because some parts of the stomach are not captured in the endoscope video.

### C. Mesh and Texture Generation Results

**FIGURE 5.**
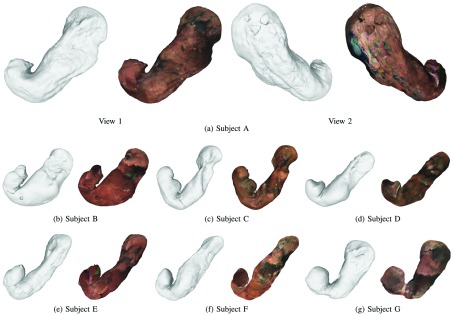
The triangle mesh and texture models generated from the final point clouds reconstructed using the red channel with the IC dye. The visible texture is the inner texture of the stomach. The video version can be seen from the following link (http://www.ok.sc.e.titech.ac.jp/res/Stomach3D/).

Figure 5 shows the results of triangle mesh and texture models generated from the final cleaned point cloud, $\tilde {\mathbb {P}}$, of the red channel with the IC dye. The visible texture is the inner texture of the stomach. We can confirm that the generated meshes represent the whole shape of a stomach for all subjects. We can also observe that local detail of the stomach such as the rugae, as can be seen in the model of Subject A, is preserved and not over-smoothed by our outlier removal algorithm. Moreover, the textured representation makes the generated 3D model more perceptible for viewers. The video version of our results can be seen from http://www.ok.sc.e.titech.ac.jp/res/Stomach3D/.

### D. Frame Localization and Local Reconstruction Results

**FIGURE 6.**
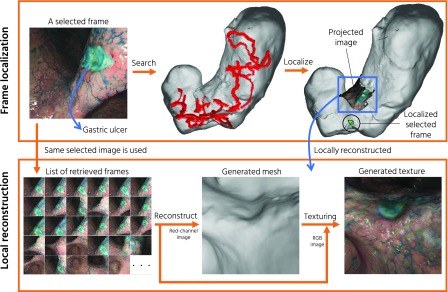
The demonstration of our frame localization and local reconstruction pipeline. Top left figure shows the selected frame containing a gastric ulcer of Subject G. The localized frame is shown as a green pyramid in the top right figure, where We also project the selected frame to the generated mesh. Using the same selected frame, we perform the local reconstruction. In addition to the detail morphological information, our pipeline provides important color texture information for easier inspection. Our pipeline is useful for identifying a particular frame’s pose within the global view of the stomach and reconstructing an area of interest.

Figure 6 shows our frame localization and local reconstruction results. As an example, we localize and reconstruct a suspected gastric ulcer in Subject G. The frame containing the ulcer is selected by a doctor as the reference frame.

The top row of Fig. 6 shows the frame localization result. Our localization pipeline localizes and projects any selected reconstructed frames (e.g., by clinicians or surgeons) to the generated triangle mesh based on the estimated endoscope poses. Our localization pipeline provides viewers with the estimated location of a particular frame, which can be used for the 3D localization of a malignant lesion.

The bottom row of Fig. 6 illustrates the process and the result of local region reconstruction. We use the ulcer image as a reference frame and retrieved its 100 closest images from all images in the corresponding sequence. The retrieved 100 images are then used as the input for the 3D reconstruction pipeline, resulting in 97 reconstructed frames. The middle and right images of the bottom row show that the local reconstruction result closely represents the actual morphological and color information which can be used for detailed diagnosis.

## IV. Discussion and Conclusion

In this paper, we have presented an SfM pipeline to reconstruct the whole shape of a stomach from a standard monocular endoscope video. For this work, we have decided to adopt SfM because it has numbers of advantages compared to other approaches such as SfS [11]–[13] and SLAM [14]–[17]. The SfS can recover the 3D structure from a single image. However, it requires accurate estimation of the light position which is a difficult problem. The SLAM approach offers real time performance required for computer-aided surgery applications. To achieve that, it uses a simple feature detector and descriptor and sequential feature matching instead of exhaustive feature matching like what we perform. These compromises lead to limited 3D reconstruction quality and completeness.

Compared to SLAM and SfS, SfM offers an off-line solution with higher reconstruction quality and completeness. SfM uses a more accruate feature detector and descriptor to obtain higher quality feature points. It also performs both local and global optimization such as bundle adjustment [30]. However, since SfM relies on the detected feature points, it is still challenging to reconstruct texture-less surfaces, which are common in internal organs. To tackle this challenge, structured light endoscope systems [37], [38] exploit an active projector to project a structured light pattern on the texture-less surfaces. Although these systems can successfully increase the number of feature points for SfM, they require expensive hardware modification.

On the other hand, we have exploited a common IC dye spraying procedure to increase the number of extracted future points without needing any hardware modification. We also have investigated the combined effect of the IC dye presences and color channel selection. Based on the result presented in Table 1, it is shown that the IC dye is able to increase the number of extracted feature points by a large margin. In addition, we have found that red channel images under the chromo-endoscopy using the IC dye provides the most complete point cloud result. For comparison, we run the base version of SLAM [39] applied in [17] on Subject B. Even on the red channel with the IC dye, the SLAM cannot obtain enough feature matches to maintain the feature tracking, resulting in the incomplete 3D model far from the whole stomach with very few reconstructed images.

We also have presented a local plane fitting-based outlier removal algorithm to clean the initial SfM result and demonstrated that our algorithm is able to effectively remove outliers from an initial SfM result and produce a clean point cloud while preserving the structure and detail of the stomach. We also have demonstrated that high-quality mesh could be obtained from the cleaned point cloud. Since our approach does not add any structured light patterns that may overlay any important medical information, we can directly use the captured images to texture the obtained mesh. Thus, the 3D model of a stomach with vital color information can be obtained from a standard gastrointestinal endoscope video. This is a novel imaging modality of gastrointestinal tract because it contains both whole morphological and color information at the same time. Even if the indicated lesion is on a flat region, it could be recognized from the color information more easily than the commonly used double contrast barium radiography [1] and the recently proposed 3D CT scan [2]. Gastric surgeons may intuitively recognize the location of the indicated lesion relative to the whole stomach, which provides a significant advantage to decide the needed operative procedures, such as total or partial gastrectomy for gastric malignancies.

As a potential application, we have demonstrated a frame localization pipeline that can visualize the estimated location of the particularly selected endoscopic video frame onto the reconstructed 3D model, which can make lesion identification more handy. We have also presented a local reconstruction pipeline that reconstructs the local region around the particularly selected frame, which provides more precise and detailed shape information. It might be applicable to the evaluations of mucosal extension of the early gastric cancer or detailed lesion type classification as performed in [20].

Our future work will be focused on the clinical significance of our proposed method. We will try to evaluate the clinical usefulness of this method for the patients undergone surgery for early gastric cancers. Application of monocular depth estimation for endoscope images [40], [41] may be one of the future directions to improve the local reconstruction.
